# Theoretical and experimental study of the “superelastic collision effects” used to excite high-g shock environment

**DOI:** 10.1038/s41598-023-29538-4

**Published:** 2023-02-09

**Authors:** Zhengyong Duan, Qihang Zeng, Dayong Tang, Yingchun Peng

**Affiliations:** Chongqing University of Arts and Sciences, 319 Honghe Avenue, Yongchuan District, Chongqing, 402160 China

**Keywords:** Engineering, Materials science

## Abstract

The excitation technology for high-g-level shock environment experiments is currently a topic of interest, for which velocity amplification by collisions of vertically stacked bodies has been used to develop high-g shock tests with great success. This study investigated the superelastic collision effects generated during high-velocity one-dimensional three-body impacts. Theoretical formulae were derived in brief for an analytical investigation of the collisions. Four experiments were performed with different initial velocities obtained from free-falls from different heights. Velocity gains larger than 5 were obtained for the three-body collisions, and coefficients of restitution larger than 2.5 were observed for the second impact. The experimental results well verified the existence of superelastic collision effects in the one-dimensional three-body impacts.

## Introduction

To maximize the damage and effectiveness of advanced penetrating weapons, such as ordnance penetrators, a smart fuse or missile-borne recorder is used to sense environmental information and control the burst point when striking the target. During the striking process, the components of such systems and the systems themselves generally experience shocks equal to tens of thousands of g (1 g = 9.8 m/s^2^) for durations of several milliseconds. All components and the systems themselves must survive such shock loading events and be qualified for severe environments^1–4^. Therefore, it is undoubtedly essential to assess the survivability and the working performance of the components and systems by testing them in such high-g shock environments during the development and production processes.

At present, high-g shock tests can be divided into two categories: laboratory tests and live ammunition tests. A live ammunition test, such as actually firing a projectile from a gun or mortar, can provide an appropriate test environment that is closest to the actual use environment. However, live ammunition tests are difficult to conduct and very costly ^3^; therefore, they are impractical for engineering development tasks that require countless iterations to achieve the desired results for individual components as well as for their assemblies. Various high-g shock testing methods can be adopted under laboratory conditions, including the drop table^5,6^, Machete Hammer^7,8^, Hopkinson Bar^9,10^ and gas gun methods^4,11^. These testing methods have their own advantages and limitations, which will not be repeated here. These limitations promote the development of high-g shock testing technology. As early as the 1960s, it was found that velocity amplification could be achieved through one-dimensional multi-body collisions^12^. Some detailed discussions about this issue can be found in subsequent literature^13–15^. Therefore, dual mass shock amplifiers (DMSAs), combined with conventional drop tables, have been given increasingly more attention for use in high-g shock testing. They claim a range of obtainable accelerations during drop testing from 5000 g to as much as 100,000 g by using secondary impacts^16–19^. However, their limitations are also obvious because they use conventional impact tables. In addition, Rodgers et al.^20–24^ developed a four-mass vertically stacked shock amplifier. However, their test results revealed that the four-mass vertically stacked shock machine did not have any advantages over the Hopkinson Bar or even the drop table methods. To date, generating various high-g shock testing environments with good reliability, repeatability, convenience, and low cost is a long-standing problem with significant difficulties. Driven by the technical demands of high-g shock tests and inspired by existing ideas, a compact high-g shock tester with a three-body vertically stacked shock amplifier was developed by the current authors. The experimental results confirmed that this design was successful^25–27^. However, a detailed study of the velocity amplification seems to have been deliberately ignored, most likely because the primary focus was on the shock acceleration pulse parameters.

The objective of this work is to further probe the subtlety behind the success of this design. The superelastic collision effects involved in the one-dimensional three-body collisions were examined specifically, both theoretically and experimentally.

## Configuration and theory

**Figure 1:**
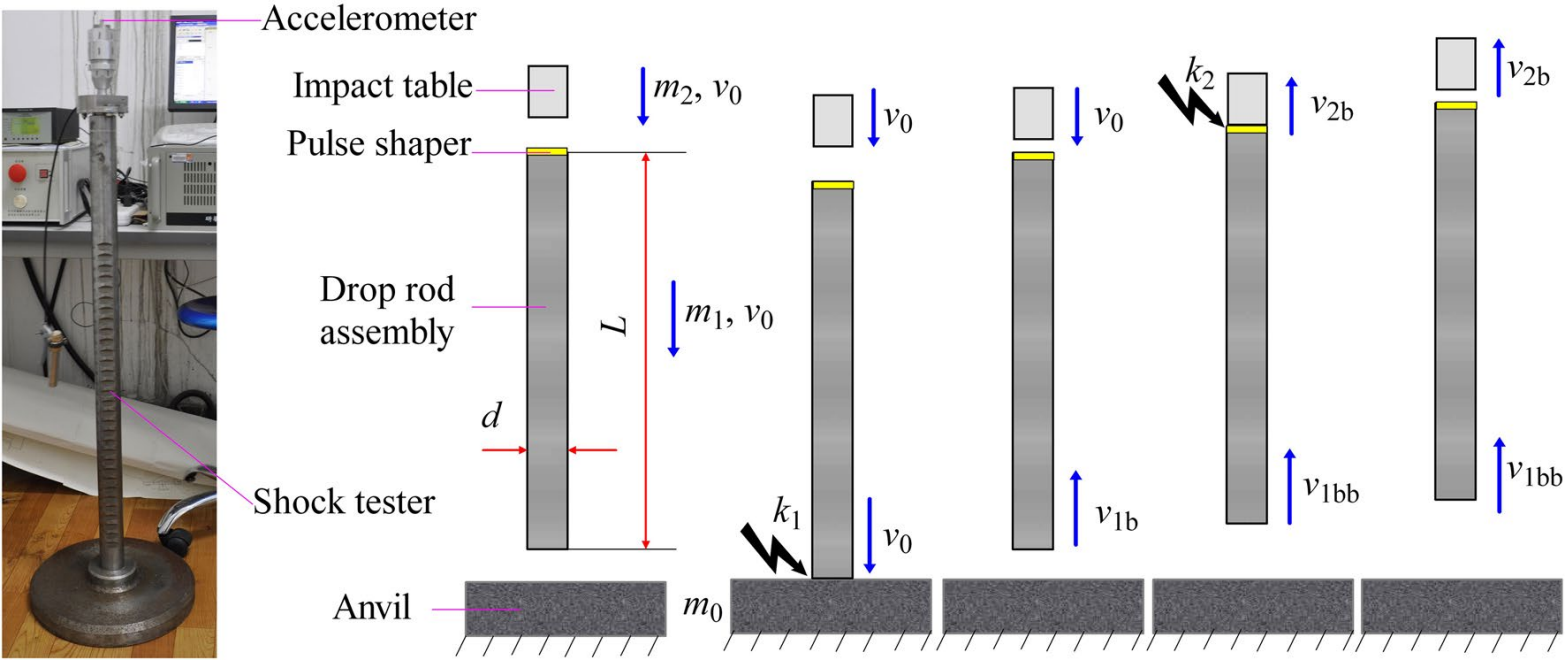
High-*g* shock tester and model of a one-dimensional three-body collision (*m*_0_ > *m*_1_ > *m*_2_). *m*_1_, *L*, and *v*_0_ represent the mass, length, and initial velocity of the drop rod assembly, respectively, *m*_2_ and *v*_0_ are the mass and initial velocity of the impact table, respectively, and *m*_0_ is the mass of the anvil. The diameters of *m*_1_ and *m*_2_ are both *d*, *v*_1b_ is the rebound velocity of *m*_1_ after striking *m*_0_, *k*_1_ and *k*_2_ are the stiffness coefficients of equivalent impact springs when *m*_1_ strikes *m*_0_ and *m*_2_ collides with *m*_1_, respectively, and *v*_1bb_ and *v*_2b_ are the velocities of *m*_1_ and *m*_2_ after *m*_2_ collides with *m*_1_, respectively.

It is well known that acceleration is defined as the rate of change of velocity with time. This provides a hint for the development of a high-g shock tester. As presented in the authors’ earlier publications, the core operating principle of their high-g shock tester was based on one-dimensional three-body collisions. The schematic and model for a one-dimensional three-body collision is shown in Fig. 1.

It is known that the coefficient of restitution describes the ratio of the relative velocities of two bodies after a collision to their relative velocities before the collision. Figure 1 shows that the anvil is fixed. If the coefficient of restitution when *m*_1_ strikes *m*_0_ is *e*_1,0_, then the rebound velocity of *m*_1_ is given by

1 $$v_{1b} = - e_{1,0} v_{0}$$

In Eq. (1), it is assumed that the velocity is positive when a particle moves upward.

Then, *m*_1_ impacts the approaching *m*_2_. Conservation of momentum yields the following equation:

2 $$m_{1} v_{1b} - m_{2} v_{0} = m_{1} v_{1bb} + m_{2} v_{2b}$$

The definition of the coefficient of restitution yields the following equation:

3 $$e_{2,1} = \frac{{v_{2b} - v_{1bb} }}{{v_{0} + v_{1b} }}$$

For a perfectly elastic collision, the conservation of kinetic energy yields the following equation:

4 $$\frac{1}{2}m_{1} v_{1b}^{2} + \frac{1}{2}m_{2} v_{0}^{2} { + }E_{p} = \frac{1}{2}m_{1} v_{1bb}^{2} + \frac{1}{2}m_{2} v_{2b}^{2}$$

Introducing the mass ratio *r*_2,1_ = *m*_2_/*m*_1_ and combining Eqs. (1)–(4) yield the following equations:

5 $$v_{1bb} = \frac{{e_{1,0} - r_{2,1} \left( {1 + e_{2,1} + e_{1,0} e_{2,1} } \right)}}{{1{ + }r_{2,1} }}v_{0}$$

6 $$v_{2b} = \frac{{\left( {e_{1,0} + e_{2,1} + e_{1,0} e_{2,1} } \right) - r_{2,1} }}{{1{ + }r_{2,1} }}v_{0}$$

The velocity gain of mass *m*_2_ after the impact is expressed as follows:

7 $$G_{2} = \left| {\frac{{v_{2b} }}{{v_{0} }}} \right| = \left| {\frac{{\left( {e_{1,0} + e_{2,1} + e_{1,0} e_{2,1} } \right) - r_{2,1} }}{{1{ + }r_{2,1} }}} \right|$$

Equations (6) and (7) suggest that larger values of *e*_1,0_ and *e*_2,1_ and a smaller value of *r*_2,1_ should yield a higher high-*g* shock environment for *m*_2_.

It is known that the value of the coefficient of restitution is equal to one if a collision is perfectly elastic and zero if a collision is perfectly inelastic. This fact suggests that for any dissipative collision that falls between perfectly inelastic and perfectly elastic, the coefficient of restitution lies between zero and one. Even assuming a perfectly elastic collision, the maximum velocity gain tends to be equal to three and is limited by *r*_2,1_ and increases as *r*_2,1_ decreases. One case of real design information for the three bodies is presented in Table 1. For this case, *r*_2,1_ = *m*_2_/*m*_1_ = 0.191 and *G*_2_ = 2.359 for perfectly elastic collisions. The directions of *v*_1bb_ and *v*_2b_ from Eqs. (5) and (6) are usually both upward in this design.

**Table 1:** Real design information for the three bodies.

Part name	Material	Density (kg/m^3^)	Young’s modulus (GPa)	Poisson's ratio	Mass (kg)	
Impact table	1Cr18Ni9Ti	7850	202	0.30	*m*_2_	0.224
Shock rod	7A09	2700	70	0.30	*m*_1_ (shock rod assembly)	0.848
Up-stand	7A09	2700	70	0.30		0.220
Cover	7A09	2700	70	0.30		0.105
Anvil	45	7850	209	0.30	*m*_0_	23.948

**Figure 2:**
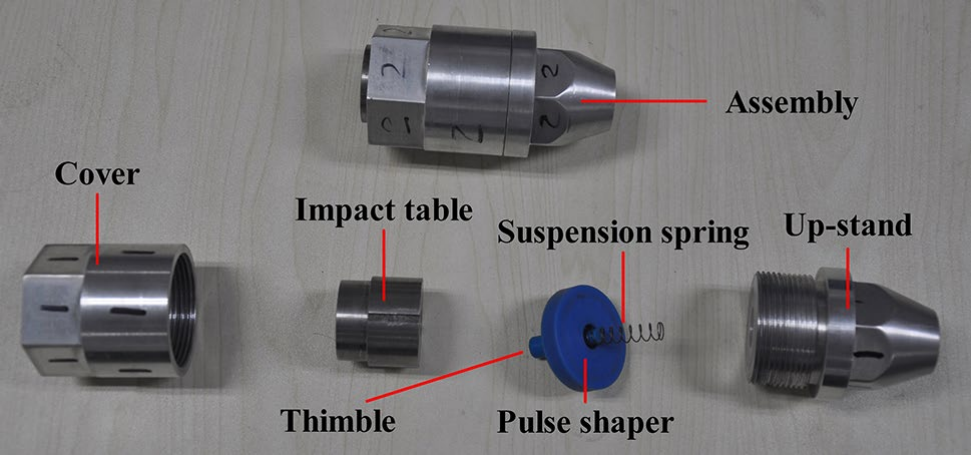
Configuration of the suspension spring and other parts.

To cause a collision between *m*_2_ and *m*_1_, a certain gap between *m*_2_ and *m*_1_ is necessary, and it must be ensured that *m*_2_ does not collide with *m*_1_ before *m*_1_ rebounds. Therefore, an elaborate suspension spring was selected to accomplish this objective. Its configuration is shown in Fig. 2.

**Figure 3:**
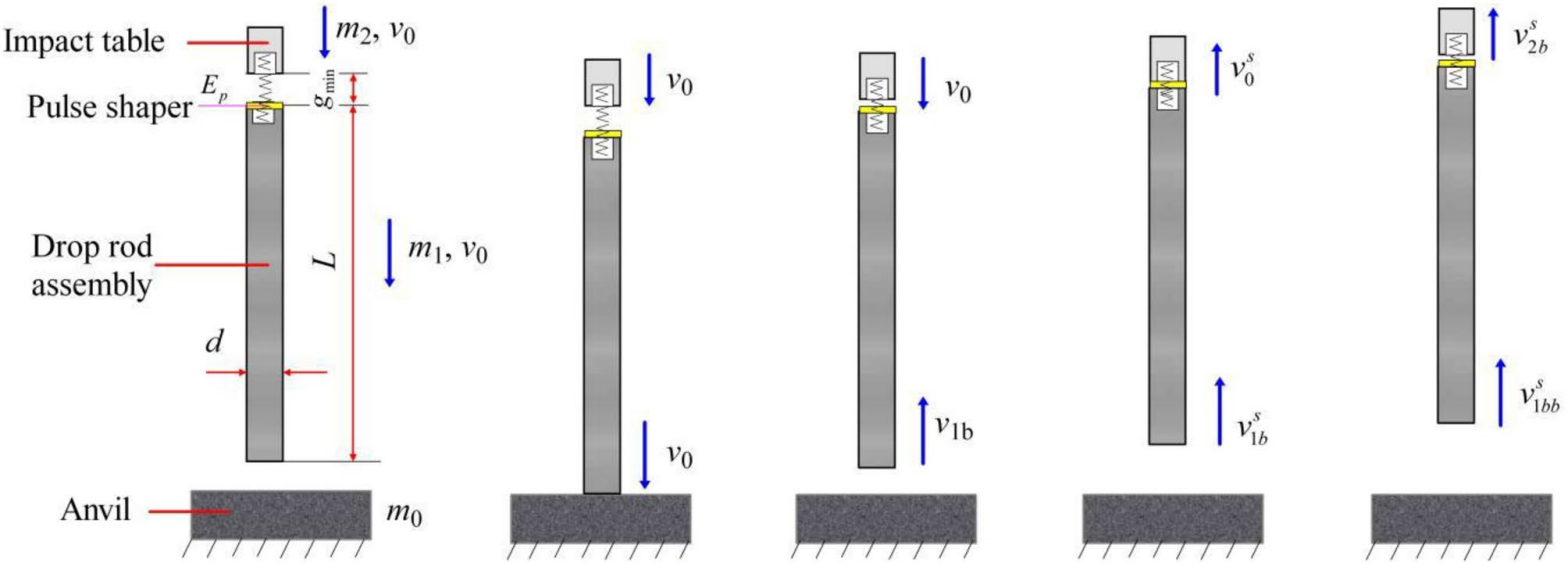
Model for a one-dimensional three-body collision that includes a suspension spring. In the figure, *E*_p_ represents the initial elastic potential energy of the suspension spring before the collision, *g*_min_ is the required minimum suspension gap, $v_{0}^{s}$ and $v_{1b}^{s}$ are the velocities of *m*_1_ and *m*_2_ before the collision, respectively, and $v_{2b}^{s}$ and $v_{1bb}^{s}$ are the velocities of *m*_1_ and *m*_2_ after the collision, respectively.

Accounting for the effect of the suspension spring, the model for a one-dimensional three-body collision is transformed into the form shown in Fig. 3.

It must be noted that the compression displacement of the suspension spring is *g*_min_ before *m*_1_ strikes *m*_2_, implying another elastic potential energy, which can be denoted as *E*_*g*_. Of course, this additional elastic potential energy will slightly influence the initial velocities of *m*_1_ and *m*_2_. Therefore, $v_{0}^{s}$ is slightly less than $v_{0}$, and $v_{1b}^{s}$ is also slightly less than $v_{1b}$.

However, the total elastic potential energy, *E*_*s*_ (*E*_*s*_ = *E*_*p*_ + *E*_*g*_), of the suspension spring certainly performs work on *m*_1_ and *m*_2_ at the moment of separation following the collision. Then, $v_{1bb}^{s}$ will be slightly less than $v_{1bb}$, but intuitively, $v_{2b}^{s}$ may be significantly larger than $v_{2b}$ because the direction of $v_{1bb}^{s}$ is upward. In essence, because the energy outside the collision system acts on the collision objects, there are superelastic collision effects, and the collision can be referred to as a superelastic collision. This implies that *e*_2,1_ is larger than one and that the velocity gain, *G*_2_, may be ecstatic.

## Experimental results and discussion

**Figure 4:**
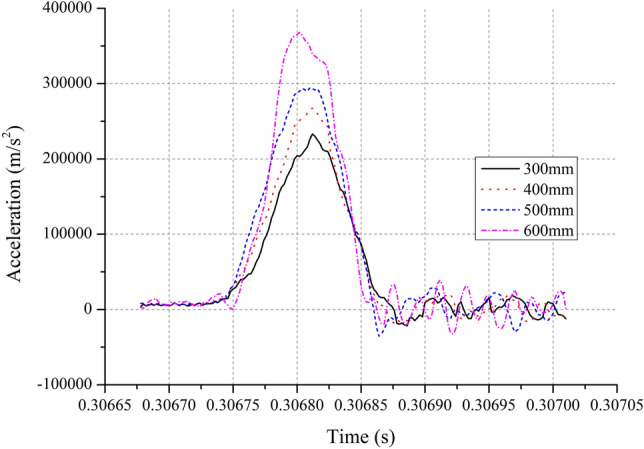
Acceleration–time curves of tests.

**Figure 5:**
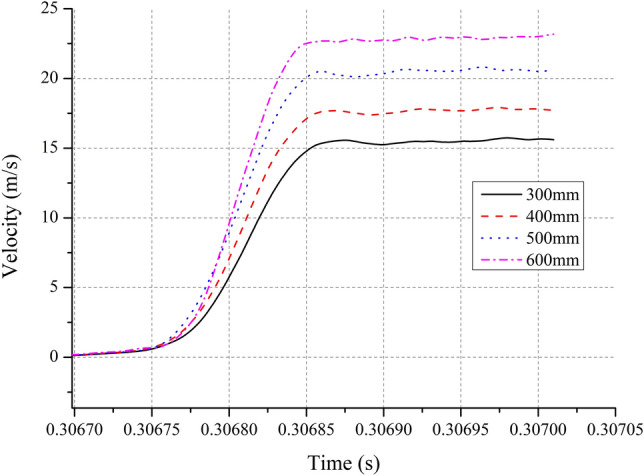
Velocity–time curves of tests.

The test configuration is shown in Fig. 1. For a 7-mm-thick PA6 pulse shaper (used to adjust the shock acceleration, pulse width and pulse waveform) and a 7A09 shock rod (*L* = 1000 mm, *d* = 20 mm), tests were conducted with various initial velocities obtained from the free-fall motion of *m*_1_ and *m*_2_ from preset heights of 300 mm, 400 mm, 500 mm, and 600 mm. By neglecting both friction and air resistance, it is deduced that the corresponding values of the initial velocities (listed in Table 2) are approximately 2.425 m/s, 2.800 m/s, 3.130 m/s, and 3.429 m/s, respectively. Acceleration–time curves were obtained by a B&K 8309 accelerometer (Fixed on the impact table with bolt) and a B&K 2692–0S1 charge amplifier, as well as by an Advantech 610 L data acquisition card, a computer, software, and a monitor. Figure 4 shows the measured acceleration–time curves, and integrating the acceleration time history into velocity^28,29^, we can obtain the corresponding velocity–time curves shown as Fig. 5.

**Table 2:** Some parameters of the tests and the theoretical analysis.

*v*_0_ (m/s)	*a*_*p*_ (m/s^2^)	$\tau$ ($\mathrm{\mu s}$)	$\Delta v$_*r*_ (m/s)	$\Delta v$_*i*_ (m/s)	$\frac{{\Delta v_{r} - \Delta v_{i} }}{{\Delta v_{i} }} \times 100\%$	$$\begin{gathered} \mathop {}\limits^{{}} v_{2b}^{s} \hfill \\ (m/s) \hfill \\ \end{gathered}$$	*G*_2_ (Eq. (7))	*e*_2,1_ (for *e*_1,0_ = 1) (Eq. (3))
− 2.425	225,607	107	14.702	15.368	− 4.333	12.277	5.063	2.610
− 2.800	260,272	106	16.815	17.564	− 4.262	14.015	5.005	2.576
− 3.130	287,387	105	19.791	19.210	3.022	16.661	5.323	2.765
− 3.429	359,233	91	21.668	20.811	4.117	18.239	5.319	2.763

Figure 4 shows that the shapes of the measured acceleration–time curves which are all closed to half-sine curves. Using the recommended methods for determining peak acceleration amplitudes and pulse widths according to MIL-STD-810G^29^ standards, referring to Fig. 4, we can determine the peak acceleration amplitudes and pulse widths. The peak acceleration amplitudes are 225,607 m/s^2^, 260,272 m/s^2^, 287,387 m/s^2^, and 359,233 m/s^2^, and the corresponding shock pulse widths are 107 $\mathrm{\mu s}$, 106 $\mathrm{\mu s}$, 105 $\mathrm{\mu s}$, and 91 $\mathrm{\mu s}$, respectively.

From Fig. 5, it is known that the stage where the velocity–time curves change rapidly is during the process of *m*_2_ colliding with *m*_1_. Corresponding to the collision start and end times determined from the acceleration history, the velocity change (denoted as $\Delta v$_*r*_) represents the difference between the corresponding times in the velocity history. The velocity changes for the four tests are 14.702 m/s, 16.815 m/s, 19.791 m/s, and 21.668 m/s, respectively. These initial velocities, results of peak acceleration amplitudes, pulse widths and velocity changes are all listed in Table 2.

It is known that for an ideal half-sine shock pulse, the velocity change can be expressed as follows:

8 $$\Delta v_{i} = \frac{{2a_{p} \tau }}{\pi }$$

where *a*_*p*_ and $\tau$ are the peak value and duration of the shock pulse, respectively.

When reconsidering the working principle of the shock tester, it is very obvious that a perfectly elastic collision, not to mention a superelastic collision, between *m*_1_ and *m*_0_ is impossible, meaning that *e*_1,0_ < 1. From a combination of the theory and test results presented previously, some parameters are determined and also listed in Table 2 for easier analysis, where *e*_1,0_ = 1.

It is noteworthy that the relative errors associated with the velocity changes are all less than ± 5% for the four tests. This proves that the method of determining the velocity change by integrating acceleration time history into velocity is effective. Additionally, it is observed that the velocity gains of mass *m*_2_ after the impacts are all larger than 5 and that the coefficients of restitution while *m*_2_ collided with *m*_1_ are all larger than 2.5. Considering that *e*_1,0_ < 1, *e*_2,1_ should be slightly larger than the values listed in Table 2. These results go far beyond the case of a perfectly elastic collision, indicating that the collisions between *m*_2_ and *m*_1_ are all superelastic collisions. In brief, the tests results verify the qualitative conclusions of the theoretical analysis. Obviously, this represents an unexpected gain derived from using the suspension spring.

The authors believe that this work coincidentally reveals the most essential reasons for the great success of their previously developed high-*g* shock tester.

## Conclusions

This paper presents a theoretical and experimental study regarding superelastic collision effects when one-dimensional three-body collisions are used to excite a high-*g* shock environment. The investigations were conducted, and primary conclusions were drawn from the study as follows.

Theoretical formulae were derived for the second of the one-dimensional three-body collisions according to conservation of momentum, the definition of the coefficient of restitution, and conservation of kinetic energy. When accounting for a suspension spring, the qualitative analysis indicates that a larger coefficient of restitution will be achieved and highlighted the possibility of a higher high-*g*–level shock environment for *m*_2_.

Tests were conducted with initial collision velocities of 2.425 m/s, 2.800 m/s, 3.130 m/s, and 3.429 m/s. Measured acceleration–time curves and velocity–time curves obtained by integrating the acceleration data were plotted. Then, peak acceleration, duration, and velocity change values were accurately extracted. The tested velocity gains are all larger than 5 for *m*_2_ and the relative errors between the experimental and the theoretical values for the velocity change are less than ± 5%. The most important result is that the coefficients of restitution for the second collision are all larger than 2.5 when the first collision is perfectly elastic, which well verified the conclusion of theoretical analysis. From the perspective of the theoretical analysis and the experimental results, it is confirmed that superelastic collision effects play a crucial role in the authors’ previously developed high-*g* shock tester.

## Data Availability

All data generated or analysed during this study are included in this published article and its supplementary information files.
